# Mediators of Pruritus in Psoriasis

**DOI:** 10.1155/2007/64727

**Published:** 2007-12-26

**Authors:** Adam Reich, Jacek C. Szepietowski

**Affiliations:** ^1^Department of Dermatology, Venereology and Allergology, Wroclaw Medical University, Ul. T. Chalubinskiego 1, Wroclaw 50-368, Poland; ^2^Institute of Immunology and Experimental Therapy, Polish Academy of Science, Rudolfa Weigla 12, Wroclaw 53-114, Poland

## Abstract

The pathogenesis of pruritus in psoriasis remains unclear. Many possible mediators were implicated to transmit or modulate this sensation in psoriasis, but none has been clearly proven to be a causative agent of itching. The most often discussed theory mentioned the importance of impaired innervations and neuropeptides imbalance in psoriatic skin. Other possible causes of itching might be increased expression of interleukin 2 or vascular abnormalities. Recent data indicated that pruritus could be also evoked by opioid system, prostanoids, interleukin 31, serotonin, or proteases. Whether these mechanisms are also involved in pruritus accompanying psoriasis requires further investigation. Limited knowledge of pruritus origin in psoriasis is responsible for the lack of the effective antipruritic treatments for psoriatics. Here, we summarize the current knowledge about the pathogenesis of pruritus in psoriasis and point out possible directions of future studies aiming the pathogenesis of this symptom in psoriasis.

## 1. INTRODUCTION

Local inflammatory mechanisms may induce pruritus in many dermatoses. Mild-to-severe pruritus accompanies numerous inflammatory skin disorders including atopic dermatitis, eczema, psoriasis, or lichen planus. Psoriasis is one of the most common chronic inflammatory skin diseases with a complex, multifactorial, and still not fully understood etiopathogenesis. The main factors contributing to the development of psoriatic lesions are genetic predispositions and immunological disturbances [1, 2]. However, the exacerbation of psoriasis can also be provoked by numerous exogenous factors including stress, smoking, infections, and some drugs [2]. Pruritus is observed in about 70 to 90% of patients with psoriasis [3–9], and many of them (at least 30%) had generalized itching [5, 6]. The mean intensity of this symptom assessed according to 10 point Visual Analogue Scale ranged between 3.7–6.4 points [5,7, 10–12]. This is less than the intensity of pruritus observed in atopic dermatitis or uremic pruritus [13, 14]. However, despite less intensive, pruritus was mentioned by many psoriatic patients as the most bothersome symptom of psoriasis [5] and it was clearly documented that pruritus intensity significantly correlated in psoriatics with degree of quality of life impairment, level of stigmatization, as well as the presence and severity of depressive symptoms [12]. It seems that patients with pruritus suffer from more severe psoriasis [4, 6, 8] although some authors did not find a significant relationship between pruritus intensity and psoriasis severity [5]. The presence and intensity of itching were independent on age, gender, marital status, family history of psoriasis or atopy, type of psoriasis, alcohol or smoking habits, duration of the disease, as well as duration of the last outbreak of psoriasis [5, 6, 8]. Despite the high frequency of this symptom, the pathogenesis of pruritus in psoriasis remains unclear. Here, we reviewed the available literature data on this symptom in order to summarize our current knowledge of the origin of pruritus in psoriasis.

## 2. HISTAMINE

Histamine, one of the major mediators of pruritus, does not seem to be involved in its development in psoriasis. There was no correlation between pruritus intensity and histamine plasma level in psoriasis, as well as no difference was observed in histamine plasma levels between pruritics and nonpruritics patients with psoriasis [10]. In addition, less than 20% of psoriatic subjects claimed that oral antihistaminics were effective in reducing pruritus [6]. It seems that only sedating antihistaminics should be tried in pruritic psoriatics as they sometimes could be effective due to evoked sedation [15]. It is generally accepted that the histamine blockade does not prevent pruritus in psoriasis [15].

## 3. NEUROPEPTIDES AND ALTERED CUTANEOUS INNERVATIONS

The most often discussed theory on pruritus in psoriasis mentioned the importance of impaired innervation and neuropeptides imbalance in psoriatic skin. Interactions between nerves, neuropeptides, and mast cells, leading to neurogenic inflammation, have also been implicated in another chronic itchy immunodermatosis: atopic dermatitis [16, 17]. Several studies demonstrated altered expression and/or distribution of several neuropeptides and their receptors within various layers of psoriatic skin, including substance P (SP), calcitonin gene-related peptide (CGRP), vasoactive intestinal peptide (VIP), somatostatin, *β*-endorphin, or pituitary adenylate cyclase activating polypeptide (PACAP) [17–26]. Neuropeptides degranulate mastocytes, activate dendritic cells, lymphocytes, macrophages, and neutrophils, and produce vascular changes in the skin by inducing angiogenesis, dilatation of vessels, and stimulation of synthesis of nitric oxide [26]. They also stimulate synthesis and release of many proinflammatory cytokines from mast cells, lymphocytes, dendritic cells, fibroblasts, and keratinocytes, induce expression of vascular adhesion molecules on endothelium, and exert hyperproliferative effect on keratinocytes [26]. Neuropeptides in the skin may be released from dermal nerve endings, but they can also be directly produced by several cell types, for example, mastocytes [17].

Nakamura et al. [27] observed that pruritic psoriatic skin demonstrated significantly increased number of nerve growth factor- (NGF-) immunoreactive keratinocytes, elevated NGF content in the lesional skin, and enhanced expression of high-affinity receptor for NGF (Trk-A) in the epidermis and dermal nerve fibres. Moreover, pruritic skin showed increased number of protein gene product (PGP) 9.5-immunoreactive nerve fibers in the epidermis and in the upper dermal areas, increased number of SP-containing nerves in the perivascular areas, as well as decreased expression of neutral endopeptidase (NEP) in the epidermal basal layer and in the endothelia of blood vessels [27]. The pruritus intensity correlated with the number of PGP 9.5-immunoreactive intraepidermal nerve fibers, the number of NGF-immunoreactive keratinocytes and the expression level of TrkA in the epidermis [27]. Nakamura et al. [27] also found an increased number of mast cells in the papillary dermis of pruritic psoriatic skin among the various cellular components examined, including resident cells and infiltrating cells in the skin lesions. Ultrastructural examination showed that these mast cells possessed degranulating specific granules indicating that mast cells in pruritic psoriatic skin are activated. The particularly characteristic finding of mast cells in lesional skin from patients with pruritus was the presence of free mast cell granules in close apposition to the perineurium surrounding unmyelinated nerve fibers. This phenomenon was never observed in the skin from patients without pruritus [27]. In contrast, Nakamura et al. [27] did not find any differences between pruritic and nonpruritic psoriatics regarding the skin expression of brain-derived neurotrophic factor, neurotrophin-3, VIP, neuropeptides Y (NPY), somatostatin, low-affinity receptor for NGF, and angiotensin-converting enzyme. In another study [8], a hyperproliferation of small cutaneous nerves was found in the lesional skin of pruritic psoriatic subjects compared to nonpruritic ones. Keratinocytes in the psoriatic plaques of patients with pruritus also showed consistently increased expression of SP receptor, TrkA and CGRP receptor, but the immunoreactivity for SP, CGRP, VIP, and PACAP was independent on the occurrence of pruritus. The expression of NGF, neurotrophin-4, low-affinity receptor for NGF, PACAP receptor expression, as well as NEP activity did not differ between pruritus and nonpruritus group [8]. Interestingly, Remröd et al. [11] did not find any relationship between SP-positive fibers nor cells and the degree of pruritus, but the analyzed group of patients in this study was very small. In addition, the NPY plasma level was significantly decreased in patients with pruritus compared to patients without pruritus [9]. Plasma levels of SP, CGRP, and VIP did not differ significantly between pruritics and nonpruritics, however, a tendency to lower SP and VIP plasma levels in patients with pruritus was noted [9]. Moreover, significant, negative correlations between pruritus severity and SP as well as VIP plasma levels were found [9]. It seems probable, that increased expression of neuropeptides in the pruritic skin might activate the neuropeptides degrading enzymes like NEP or angiotensin-converting enzyme in a regulatory mechanism. This phenomenon could lead to the decreased plasma level of selected neuropeptides. This hypothesis could be supported by the observations that the proportion between chymase- and tryptase-positive mast cells was shown to be disturbed in lesional psoriatic skin [28] as well as patients with psoriasis were characterized by higher serum activity of angiotensin-converting enzyme which was normalized after effective antipsoriatic treatment [29]. In the study by our group [10] it was noted that CGRP plasma level was significantly elevated in pruritic psoriatic patients compared to healthy subjects, a difference that was not found between nonpruritic psoriatics and healthy volunteers, and that CGRP plasma level correlated with itching intensity in some subgroups of psoriatics. The important role of altered innervations and neuropeptide imbalance in pruritus accompanying psoriasis may also be supported by the observations that topically applied capsaicin, a potent SP depletory, effectively treated pruritus in psoriatics [30, 31]. Finally, it was documented that stress-exacerbated pruritus in psoriasis [7] and neuropeptides seem to be good candidates for linking nervous system and skin [17]. It could be hypothesized that increased innervations in the skin of psoriatic patients with pruritus may lead to a lower threshold for pruritic stimuli compared to patients without pruritus. Additionally, pruritus might be evoked by the release of selected neuropeptides from dermal nerve endings and cells during stress, but this hypothesis still requires further investigations (Table 1).

## 4. CYTOKINES

Concerning the role of cytokines in pruritus in psoriasis, Nakamura et al. [27] found an increased number of interleukin (IL)-2 immunoreactive cells in pruritic versus nonpruritic lesions of psoriasis (Table 1). There were no significant differences in the expression of other cytokines (interferon (INF)-*γ*, tumor necrosis factor (TNF)-*α*, IL-1*α*, IL-1*β*, IL-4, IL-5, IL-6, IL-8, IL-10, and IL-12) [27]. Recently, a novel cytokine, IL-31, was suggested to play an important role in pruritus in atopic dermatitis, as IL-31 caused the itch-associated scratching behavior in conventional NC/Nga mice, an experimental animal model for atopic dermatitis [32]. Whether this cytokine also participates in pruritus in psoriasis needs to be determined.

## 5. VESSELS AND ADHESION MOLECULES

Vascular abnormalities are frequently observed in psoriatic lesions [33]. It seems that changes of dermal vasculature may be important in the pathogenesis of pruritus in psoriasis (Table 1). A marked increase of the density of E-selectin-positive venules was found in psoriatic patients with pruritus compared to nonpruritic subjects [27]. However, there was no statistical difference in the number of vessels immunoreactive for intercellular cell adhesion molecule (ICAM)-1, vascular cell adhesion molecule (VCAM)-1, or platelet endothelial cell adhesion molecule (PECAM)-1 in the upper dermis or in the expression of ICAM-1 in the epidermis [27]. However, significant correlation was observed between the itching intensity and the density of E-selectin-immunoreactive vessels [27]. In addition, Madej et al. [33] found an increased serum concentration of soluble vascular adhesion protein (VAP)-1 in psoriatic subjects with pruritus compared to patient free of this symptom.

## 6. OTHER POSSIBLE MEDIATORS

Despite the lack of solid laboratory data, other mediators may also play a role in the pathogenesis of pruritus in psoriasis (Table 1). They were found to be important in several pruritic conditions, but have not been investigated in psoriasis yet.

It could be speculated that neuropeptides in psoriatic skin may induce expression and/or activity of dermal proteases, and these enzymes acting via protease-activated receptors (PAR) might be responsible for prurtius [43]. Recent findings suggested that proteases are not only degrading enzymes, but rather represent a group of mediators communicating with nerves, and thereby modulating inflammation, pain, and pruritus [43, 44]. A massive itch behavior was noted in mice overexpressing epidermal kallikrein-7 [43]. Tryptase and microbial proteases induced itch by the PAR-2-mediated neurogenic mechanism [43, 45]. Activation of PAR-2 evoked itching both in mice and in human [43–46]. Because PAR-2 is irreversibly activated by proteases, it might be a good candidate for the explanation of chronic itch.

Pruritus may be elucidated by the opioid system as well. It is believed that activation of *μ*-opioid receptors induces while activation of *κ*-opioid receptors alleviates pruritus. A significantly altered *μ*- and *κ*-opioid receptor expression was observed in the epidermis of patients with atopic dermatitis, showing mainly downregulation of *κ*-opioid system [35, 36]. PUVA treatment, a frequently applied and effective therapy of atopic dermatitis, was shown to reconstitute the altered opioid receptor distribution in epidermis of these patients [36]. Opioids may also induce pruritus acting in central nervous system. It was shown that intrathecal administration of morphine elicits pruritus and both naloxone and naltrexone, the potent *μ*-opioid receptor antagonists, reduces histamine-induced pruritus in atopic dermatitis subjects to greater extend than antihistaminic drugs [37, 38]. On the other hand, nalfurafine, a *κ*-opioid receptor agonist, led to significant reduction of itching in patients with uremic or cholestatic pruritus [39, 40].

Prostanoids, mainly prostaglandin D_2_ [41, 42] and tromboxane A_2_ [47] or serotonin [48], could be further candidates as mediators of pruritus in psoriasis. The importance of the latter one might be supported by the observations that mirtazapine, an antihistaminic drug acting also via noradrenergenic *α*2-receptors and 5HT_2_ and 5HT_3_ serotonin receptors, relieved psoriatic itch even in cases of severe pruritus associated with erythrodermic psoriasis [34].

## 7. THE ROLE OF CENTRAL NERVOUS SYSTEM

Pruritus causes the desire to scratch the skin and is experienced as a sensation arising in the skin [49]. However, like all other skin sensations, itch is a product of central nervous system activities [49]. The itch-selective spinal neurons form a distinct pathway projecting from lamina I of the spinal cord to the ventrocaudal part of the nucleus medialis, which projects to the anterior cingulated and dorsal insular cortex [49]. Recent studies characterized the supraspinal processing of itch in humans by different imaging techniques. Intradermal injection of histamine in healthy volunteers led to activation of anterior cingulate cortex, supplementary motor area, premotor area, and inferior parietal lobe [50, 51]. Prolonged itch stimuli activated a superior frontal gyrus and the gyrus rectus in both hemispheres as well as in a small area of the left anterior cingulated gyrus [52]. Further activation was located in the left temporal pole and some parts of the left cerebellum [52]. Repetitive scratching induced bilateral activation of the secondary somatosensory cortex, insular cortex, inferior parietal lobe, and cerebellum while anterior and posterior cingulated cortices were deactivated [53]. The main limitation of these studies is the observations of healthy subjects. As it was demonstrated by Ishiuji et al. [54], the brain processing of itch in chronic skin conditions like in atopic dermatitis is significantly different than in healthy individuals. Therefore, further data are needed to identify the brain areas responsible for pruritus in patients with chronic itch, including those having psoriasis.

## 8. CONCLUSIONS

Summarizing, pruritus is an important symptom of psoriasis. Despite the fact that several studies have been undertaken to investigate the pathogenesis of pruritus in psoriasis, many aspects have not been studied yet (Table 1). Therefore, the pathogenesis of this symptoms is far to be well understood and, as a consequence, the therapy of pruritic psoriatic patients still remains a big challange for clinicians. We hope that in the near future new studies will be conducted to better characterize and understand this symptom in psoriasis. We do believe that this progress may facilitate the development of new effective antipruritic treatment modalities.

## Figures and Tables

**Table 1:** Possible mediators involved in itching in psoriasis.

Mediator	Comment	References
Histamine	Seems not to be involved in pruritus in psoriasis.	[6, 10]
NGF	Increased number of NGF-immunoreactive keratinocytes, elevated NGF content in the lesional skin and enhanced expression of Trk-A in the epidermis and dermal nerve fibers in psoriatics with pruritus.	[8, 27]
Substance P	Increased number of SP-containing nerves in the perivascular areas of pruritic psoriatic skin, increased expression of SP receptor in epidermis from pruritic psoriatic subjects.	[8, 27, 30, 31]
CGRP	Increased expression of CGRP receptors in pruritic psoriatic skin, increased serum level of CGRP in pruritic psoriatic subjects.	[8, 10]
NPY	Decreased NPY plasma level in psoriatic patients with pruritus.	[9]
VIP/PACAP	Negative correlation between pruritus severity and VIP plasma level	[9]
IL-2	Increased number of IL-2 immunoreactive cells in pruritic versus non-pruritic lesions of psoriasis.	[27]
IL-31	Data confirming its role in itching in atopic dermatitis; no data regarding psoriasis.	[32]
E-selectin	Increased density of E-selectin positive venules in psoriatic patients with pruritus.	[27]
VAP-1	Increased serum concentration of soluble VAP-1 in psoriatic subjects with pruritus.	[33]
Serotonin	Only indirect data suggesting its importance for pruritus in psoriasis.	[34]
Opioids	Possible mediators, but no studies in psoriasis are available.	[35–40]
Prostanoids	Possible mediators, but no studies in psoriasis are available.	[41, 42]
Proteases	Possible mediators, but no studies in psoriasis are available.	[43–46]

CGRP: calcitonin gene-related peptide, IL: interleukin, NGF: nerve growth factor, NPY: neuropeptide Y, PACAP: pituitary adenylate cyclase activating polypeptide, PARs: protease activated receptors, Trk-A: high-affinity receptor for NGF, VAP-1: vascular adhesion protein 1, VIP: vasoactive intestinal peptide.
